# Neolithic dental calculi provide evidence for environmental proxies and consumption of wild edible fruits and herbs in central Apennines

**DOI:** 10.1038/s42003-022-04354-0

**Published:** 2022-12-19

**Authors:** Alessia D’Agostino, Gabriele Di Marco, Silvia Marvelli, Marco Marchesini, Elisabetta Rizzoli, Mario Federico Rolfo, Antonella Canini, Angelo Gismondi

**Affiliations:** 1 PhD Program in Evolutionary Biology and Ecology, Department of Biology, University of Rome Tor Vergata, Rome, Italy; 2 Laboratory of Botany, Department of Biology, University of Rome “Tor Vergata”, Rome, Italy; 3 Laboratorio di Palinologia e Archeobotanica-C.A.A. Giorgio Nicoli, San Giovanni in Persiceto, Bologna, Italy; 4 Department of History, Culture and Society, University of Rome “Tor Vergata”, Rome, Italy; 5 Present Address: Laboratory of Botany, Department of Biology, University of Rome “Tor Vergata”, Rome, Italy

**Keywords:** History, Agriculture, Plant domestication, Evolutionary ecology, Biodiversity

## Abstract

Looking for a biological fingerprint relative to new aspects of the relationship between humans and natural environment during prehistoric times is challenging. Although many issues still need to be addressed in terms of authentication and identification, microparticles hidden in ancient dental calculus can provide interesting information for bridging this gap of knowledge. Here, we show evidence about the role of edible plants for the early Neolithic individuals in the central Apennines of the Italian peninsula and relative cultural landscape. Dental calculi from human and animal specimens exhumed at Grotta Mora Cavorso (Lazio), one of the largest prehistoric burial deposits, have returned an archaeobotanical record made up of several types of palaeoecological proxies. The organic fraction of this matrix was investigated by a multidisciplinary approach, whose novelty consisted in the application of next generation sequencing to ancient plant DNA fragments, specifically codifying for *maturase K* barcode gene. Panicoideae and Triticeae starches, together with genetic indicators of Rosaceae fruits, figs, and Lamiaceae herbs, suggested subsistence practices most likely still based on wild plant resources. On the other hand, pollen, and non-pollen palynomorphs allowed us to outline a general vegetational framework dominated by woodland patches alternated with meadows, where semi-permanent settlements could have been established.

## Abstract (web summary)

Next generation sequencing of plant ancient DNA and microscopic evidence from dental calculi of specimens exhumed at Grotta Mora Cavorso (Central Italy) provide insight about the role of edible plants and cultural landscape during early Neolithic.

## Introduction

The role of plant foods in pre-agrarian societies remains one of the major issues of Prehistory. Archaeobotanists are interested in looking for information about past plant biodiversity, as well as land use and exploitation, to understand how food demand and storage favoured sedentism. It is important to critically investigate the development of ancient practices for sustainable management of the territory and the use of wild plants as food, phytoremedies, textiles, pigments, fuels, etc.

Direct evidence for feeding behaviour is scant. This is because the botanical material in archaeological contexts is almost always lost and, that which survives, would need of specific molecular/genetic identification. However, recent methods applied to dental calculus have provided a wide understanding of the dietary preferences of human and animal and their interaction with the environment^1–4^.

In this context, the palynological analysis from tartar has proved an efficient tool for palaeoecological and feeding ecology reconstructions^5–7^. In fact, it is recognised that conditions of reduced aerobic degradation may promote ancient debris conservation. A fast burial creates a closed system where mineral substrates can enhance preservation, cementing subfossil cytoplasm and preventing the debris’ oxidation^8–10^. Similarly, dental calculus mineral matrix is believed to be relatively isolated from the taphonomic processes which degrade soft tissues post-mortem and, therefore, can provide a unique insight into dietary habits underrepresented by conventional approaches^11–13^. However, the incidence and preservation rate of palynomorphs in human calculus are still not well known and further studies are required.

More recently, advances in molecular biology have improved the capacity to extract DNA, proteins, and metabolites from tartar, as well as the application of next-generation sequencing (NGS) techniques (i.e., amplicon sequencing, metagenomics), promoting the identification of the biological component^4,14–17^. Regarding NGS on dental calculus, literature documents massive parallel sequencing of DNA regions amplified by using specific primers for a given species^18^ and high-throughput sequencing aimed to theoretically detect any DNA molecule embedded in the organic fraction^19,20^.

**Fig. 1:**
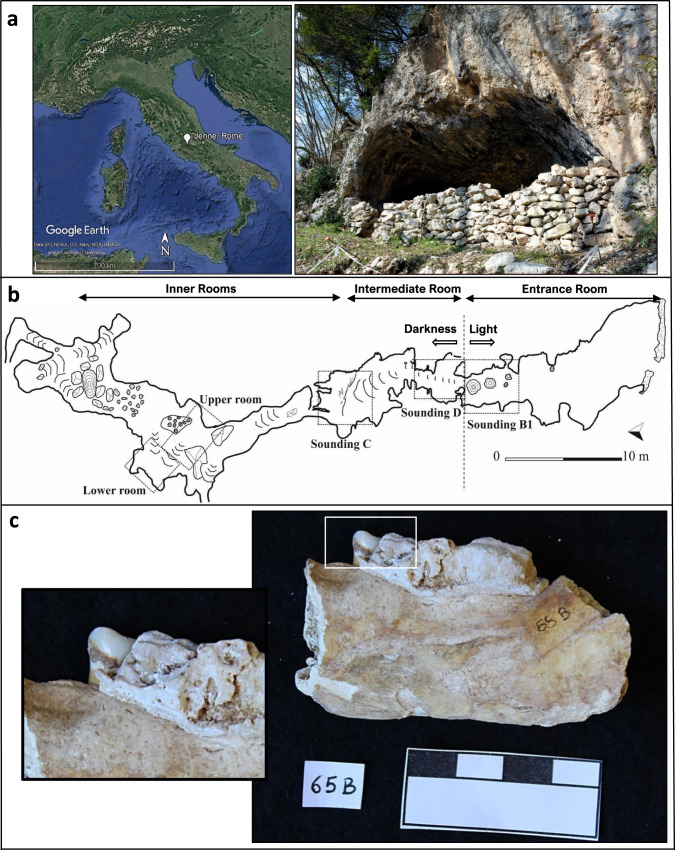
Grotta Mora Cavorso (Rome, Lazio, central Italy) and dental calculus on human remain. **a** Geographical localisation of Jenne (41° 53′ 24″ N; 13° 10′ 14″ E; Eye alt 881 m; image produced using Google Earth Pro V 7.3.3.7786, June 25, 2019; image Landsat/Copernicus; Data: SIO, NOAA, U.S. Navy, NGA, GEBCO; https://www.google.com/earth/; accessed January 2022) and image of the cave entrance captured by M.F. Rolfo. **b** Planimetry of the cave system produced by M.F. Rolfo^148^ and here redrawn. **c** Fragment of the masticatory apparatus and detail of teeth and dental calculus (images captured by Silvia De Rossi).

According to all this set of premises, the present contribution focuses on taking a comparative and integrated approach in order to study the role of plants in the subsistence practices of Neolithic individuals living in the surroundings of Grotta Mora Cavorso (Lazio, central Italy) (Fig. 1; dating in Supplementary Fig. 1). In detail, three different methodologies were applied on dental calculus samples: optical microscopy (OM), gas-chromatography mass-spectrometry (GC-MS), and NGS. For the latter method, we have specifically chosen to investigate the maturase K (*matK*) barcode gene, which is part of the chloroplast genome and currently considered as one of the most discriminating DNA regions at intra-specific and inter-specific level^21,22^. Finally, this research integrates a series of previous data obtained from archaeobotanical, palaeobotanical, anthropological, palaeontological, geological, and material culture/lithic industry analyses, exploiting the link between biological sciences and archaeology and promoting the enhancement and preservation of the cultural heritage.

## Results

Codes for human and animal dental calculus samples analysed in this work are reported in Supplementary Table 1, together with the applied method for each of them.

### Human dental calculus morphological analysis

#### Starch

Thirty-two starch granules in a good state of preservation were retrieved from 12 dental calculi out of a total of 44 and listed in Supplementary Tables 2 and 3. They were clustered in two morphological types and described using the International Code for Starch Nomenclature^23^. Shape, size, presence of *lamellae* and *hilum*, and aggregation level were the main features used for the taxonomic identification. Morphologic and morphometric parameters are described in Table 1.

**Table 1:** Proposed identification for microparticles observed in ancient dental calculi.

Starch morphotype	Morphologic and morphometric description	Proposed identification
I	The diagnostic starches were oval to sub-round in 2D shape (18–45 µm in length; 15–40 µm in width), with a central and distinct hilum and visible lamellae. The small ones (≤10 μm in diameter) were spherical in shape with a central hilum. Sometimes, the morphology was partially altered, possibly due to grinding, chewing, and/or cooking procedures	Triticeae tribe
II	Starch grains were oval to polygonal in shape, with centric hilum, perpendicular extinction cross, and evident central fissures; sometimes still lodged together. This morphotype was 13–19 μm in length and 11–17 μm in width	Panicoideae subfamily
Pollen type	Morphologic and morphometric description	
* Fagus*	Spheroidal, colporate, and isopolar pollen with long and narrow colpi. The size was 28–29 µm in length and 27 µm in width	
* Quercus* deciduous group	Prolate, isopolar, and elliptic in equatorial view (polar axis 15–26 µm long) palynomorph, and tricolpate, with pores at times indistinct	
Pinaceae (e.g., *Pinus*)	Bisaccate monad, with an elliptic corpus, medium reticulation on bladders, and dimensions of 52.5–96 µm in equatorial view, including sacci	
Cupressaceae	A non-saccate pollen, spherical (with polar and equatorial axes of 27 µm) and inaperturate. The protoplast exhibited a star-like morphology	
* Salix*	Dry tricolpate, and prolate pollen with reticulate exine sculpturing (polar axis of 20 µm; equatorial axis of 10 µm)	
Fabaceae undifferentiated	Isopolar, trizonocolporate, and prolate in equatorial view (polar axis of 24–25 µm) with reticulate exine ornamentation	
* Vitis*	Small monad, isopolar (sub-spherical to triangular shape; polar axis of 16 µm; equatorial axis of 20 µm) and tricolporate	
Poaceae	Spherical (apolar) and monoporate pollen (size: 25 µm in diameter)	
Cyperaceae	Inaperturate and pear-shaped pollen, with a scabrate sculpture (in equatorial view: triangular). The mean of the polar axis length was 43 µm	
Chenopodiaceae-Amaranthaceae	Spheroidal pollen (diameter: 16 µm) with pantoporate aperture condition	
Asteroideae undifferentiated	Isopolar, spheroidal shape, and colporate pollen with echinate ornamentation and diameter 15 µm	
Fern spore	Psilate spore (size: 23 µm in length; 14 µm in width), similar in shape to kidney beans	
Non-pollen palynomorphs	Morphologic and morphometric description	Proposed identification
Plant trichome	Multicellular and compound stellate plant hairs, characterised by 6–8 subulate branches (in length approximately 124–338 µm).	–
Capillary bristle	A fragment of capillary bristle (in length approximately 1.87 mm), barbellate throughout, from Asteraceae *pappus*	Asteroideae/Cichorioideae subfamilies
Unknown origin	Microremains with a weird swirled fibrous appearance, tangential siliceous needle-like scales, and a dimension range between 25 and 50 µm in diameter	Heliozoans/dinoflagellate cysts/resting bodies of sponges
Wood fragment	Tracheid fragment with torus-margo pit (softwood)	Conifers

Detailed description of the morphological and morphometrical parameters for each starch morphotype, pollen type, and non-pollen palynomorph retrieved from animal and human samples.

##### Morphotype I

**Fig. 2:**
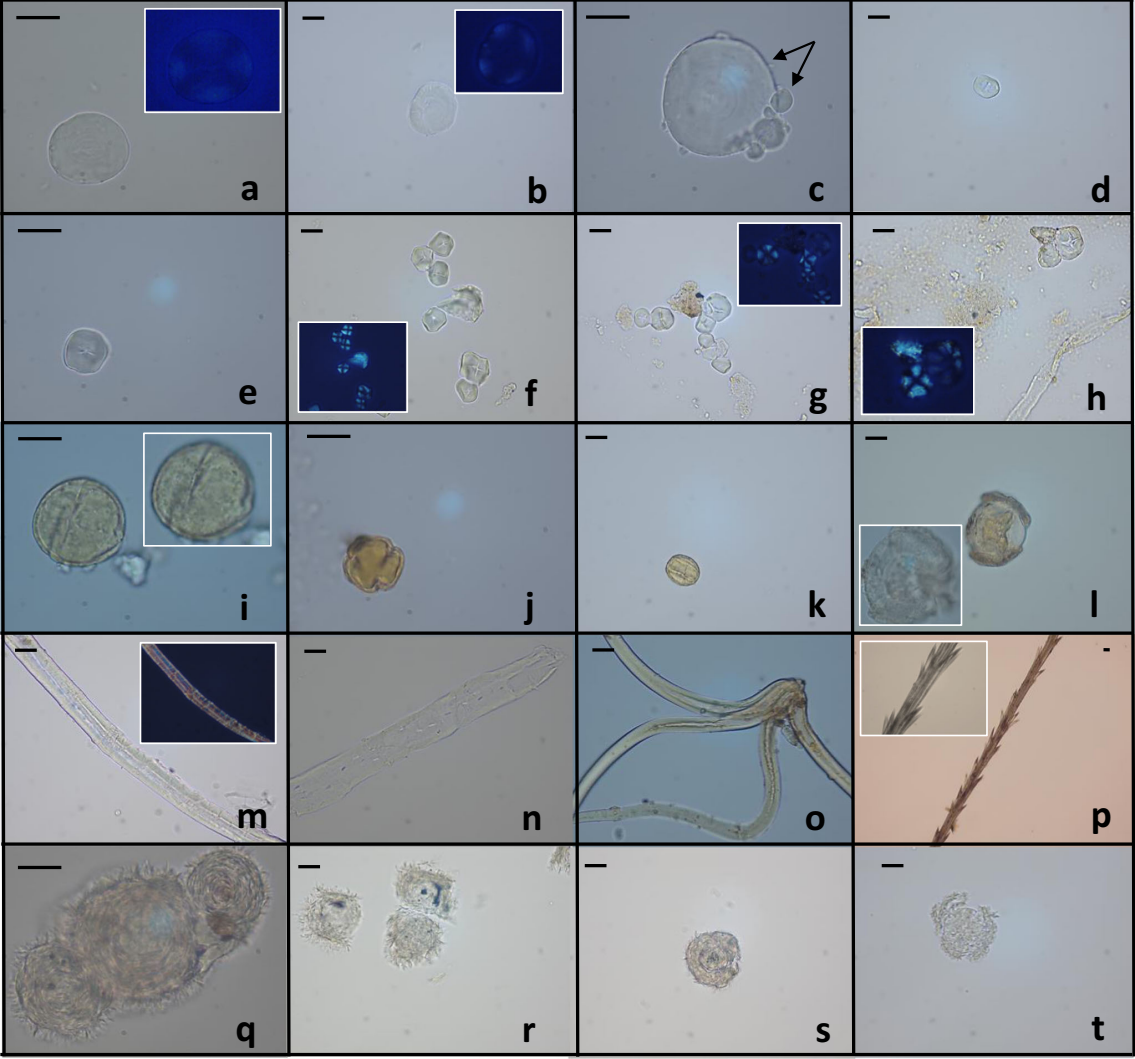
Ancient microremains from human calculi. Representative images captured by optic microscopy were shown. **a**, **b** Triticeae starches. **c** Aggregate of Triticeae starch granules, the arrows indicate the co-presence of large and small starches. **d**, **e** Panicoideae starches. **f** Aggregates of Panicoideae starch granules emerging from undissolved dental calculus. **g**, **h** Panicoideae starch granules aggregates, adhering flecks of calculus can also be seen. **i** Fagaceae, *Fagus* pollen. **j**, **k** *Quercus* deciduous pollen. **l** Pinaceae pollen, in the white box another focus of corpus and sacci is reported. **m**, **n** Plant fibres. **o** Stellate trichome. **p** Cichorieae/Astereae fruit fragment. **q**–**t** Unknown microparticles. Subpanels with dark background represent polarised images. The scale bar indicates 15 µm.

Seven samples belonging to human isolated teeth showed starch consistent with those of Triticeae Dumort. tribe (Supplementary Table 3 and Fig. 2a–c). Co-presence of large and small granules, a phenomenon known as bimodal distribution, was observed; this is a common condition in caryopses of cereals, such as *Hordeum* sp. L. and *Triticum* sp. L.^2,24^.

##### Morphotype II

Starch grains with oval to polygon (2D) shape (Fig. 2d–h) were recovered in one human sample (Supplementary Table 2) and 5 calculi from isolated teeth (Supplementary Table 3), sometimes still lodged together. This morphotype usually occurs in seeds of grasses belonging to the Panicoideae Link. subfamily^25^. Paniceae R. Br. tribe can be considered as potential candidate. Starch of species related to this tribe (e.g., *Setaria* sp. P. Beauv., common name foxtail millet, and *Panicum* sp. L., common name millet) overlap in size and shape; thus, the identification at a lower taxonomic level is arduous.

Six partially digested starches with no identifiable features, that is undiagnostic, were found in five samples. They were indicated as not determined in Supplementary Tables 2–5. Probably, salivary enzymes and bacterial activity, together with grinding process, cooking procedure in water, and/or chewing, might have altered their shape.

#### Pollen

Human calculi showed the presence of different pollen types (Supplementary Tables 2 and 3). In total, 20 pollen grains were found singly in good or excellent state of preservation. These palynomorphs (see description in Table 1) were identified according to morphometric parameters described in literature and evidenced in the Palynological Database^26^. The names of the pollen types refer to literature^27,28^. Four monads were closely like Fagaceae palynomorphs. Fagaceae pollen show a high variability in size, shape, and sculpturing, although it overlaps in morphology for some species. One of them was attributed to *Fagus* pollen type (Fig. 2i)^29^, while the other three grains to *Quercus* deciduous pollen type (Fig. 2j, k)^30^. Four microdebris displayed morphological traits consistent with Pinaceae (Fig. 2l; three in total) and Cupressaceae pollen types (one pollen)^31–33^. Two pollen types were reported although their identification remains uncertain. One was ascribed to *Salix* sp.^34^, while the other one was attributed to Fabaceae undifferentiated pollen. A small monad appeared morphologically like *Vitis* pollen^35^. Two ancient pollen grains showed a morphology which typically occurs in Poaceae species. Three Cyperaceae palynomorphs^36^ were identified in two samples. A Chenopodiaceae-Amaranthaceae pollen type^37^ was recognised in one isolated teeth sample.

Finally, three pollen grains out of 20 were not distinguishable due to the lack of diagnostic characteristics.

#### Non-pollen palynomorphs (NPPs)

Intriguing microparticles were recovered in human calculi, other than starch and pollen. They were classified as palynodebris elements and microparticles of unknown origin. Several isolated plant fibres were found in all samples (Fig. 2m, n). One calculus showed a tissue fragment consisting of a cellular structure typical of plants. The taxonomic identification of such types of microdebris is problematic due to the low preservation level and the ubiquity of these structures in the plant kingdom^38^. Two multicellular and compound stellate trichomes (description in Table 1) were embedded in two dental calculi (Fig. 2o). However, this type of trichomes occurs on the adaxial surface of the leaf epidermis of several plant species; thus, their morphology cannot be diagnostic^39^. A fragment of capillary bristle from Asteraceae *pappus* was observed in one sample (Table 1 and Fig. 2p). Usually, Asteraceae fruits are very distinct from pericarps of other families but exhibit common anatomical and morphological features among species/tribes^40^. The ancient remain could be attributed to Asteroideae/Cichorioideae subfamilies, showing similar appearance to bristles of their *pappus* (see modern reference in Supplementary Fig. 2). Nine samples revealed 14 microparticles of unknown origin (Fig. 2q–t). The morphology of these microremains (Table 1) is mainly reminiscent of sun-animalcules or heliozoans (e.g., Choanocystidae and Raphidiophryidae families), but also of dinoflagellate cysts (phylum Dinoflagellata) and/or resting bodies of sponges (known as gemmules; phylum Porifera: Spongillidae). This type of microfossils has never been observed in ancient human dental calculus. However, the visible characters are insufficient to delineate a taxonomic attribution.

### Animal dental calculus morphological analysis

#### Starch

**Fig. 3:**
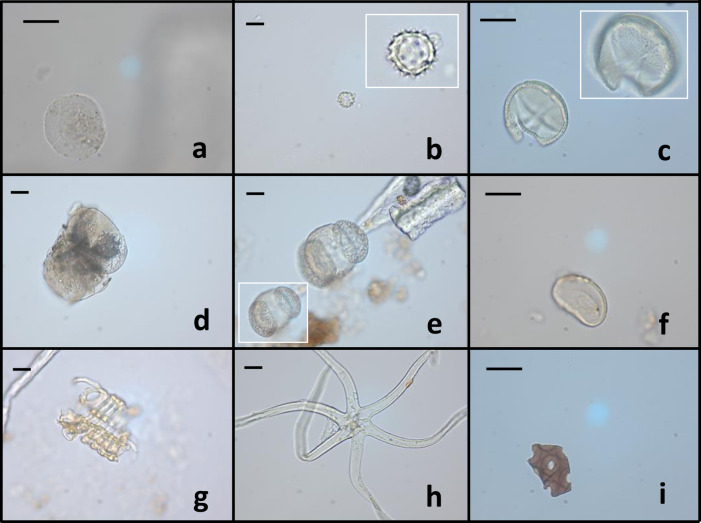
Plant microparticles from animal calculi. Representative images obtained by light microscopy analysis were shown. **a** Triticeae starch. **b** Undifferentiated Asteroideae pollen. **c** Undifferentiated Fabaceae pollen, the upper focus is reported in the white box. **d** Lump of Pinaceae pollen. **e** Pinaceae pollen, adhering flecks of calculus can also be seen, and another focus of this microremain is in the white square. **f** Monolete spore. **g** Spiral vessels. **h** Stellate trichome. **i** Tracheid fragment from conifer wood. The scale bar indicates 15 µm.

The morphological identification of granules was performed as previously described for human samples. Two animal calculi showed starches consistent with those of Triticeae tribe (Table 1, Supplementary Table 4 and Fig. 3a), while one sample revealed a gelatinised grain, maybe subjected to modification event (e.g., alpha-amylase digestion).

#### Pollen and spores

In the animal samples, six pollen were identified (Supplementary Table 4). One was attributable to Asteraceae subfamily Asteroideae undifferentiated (Table 1 and Fig. 3b). The other four grains, instead, were attributable to three pollen types already described for human samples (Table 1): Fagaceae, *Fagus* sp., Fabaceae undifferentiated (Fig. 3c), and Pinaceae, *Pinus* sp. (Fig. 3d, e). A fern spore was recognised in one sample (Table 1 and Fig. 3f). This microdebris was attributed to an indeterminate pteridophyte monolete spore, as a more specific taxonomical identification would be risky.

#### NPPs

Three spiral xylem vessels were found in one sample of *Cervus* sp. (Supplementary Table 3 and Fig. 3g), while a stellate trichome (Table 1 and Fig. 3h) and a tracheid fragment from conifer wood (Table 1 and Fig. 3i) were observed in *Martes* sp. calculus.

### Biochemical analysis

Human and animal dental calculus was subjected to GC-MS analysis and the obtained analytes were reported, for each sample, in Supplementary Data 1. Almost all chromatographic profiles revealed a series of straight-chain saturated hydrocarbons, unsaturated aliphatic hydrocarbons, and alcohols. These types of molecules would represent degradation forms of oral microbiota and/or organic matter from animal or plant debris (e.g., unsaturated, and saturated fat/oil-derived acyl lipids; higher plant waxes)^41–44^. As not univocal, they were not considered informative for residue source determination and are not included in Supplementary Data 1. Saturated and unsaturated fatty acids were the most recurrent molecules in all samples. They are widely distributed in nature (e.g., animals, plants, bacteria) at different proportion^43,45^. The ratio between hexadecanoic and octadecanoic acids was not evaluated since the organic component of tartar derives from multiple sources, simultaneously. In addition, long-chained polyunsaturated fatty acids (PUFAs; e.g., eicosapentaenoic and docosahexaenoic acids) were detected in 16 human calculi. Cyclic monoterpenes (i.e., limonene, thujanone), acyclic monoterpenoids (i.e., citronellol, linalool), bicyclic monoterpenes (i.e., camphor, myrtanol), monoterpene derivatives (i.e., eucalyptol), sesquiterpene derivatives and sesquiterpenoids (i.e., guaiene, bisabolene, santalene, calamenene, epicubebol), tetraterpenes (i.e., lycopene), and coumarins were present in some human samples. Other markers ascribable to plant material, namely one quinolizidine alkaloid (i.e., angustifoline), a pyranone (i.e., larixic acid), organic acids (i.e., tartaric acid), and plant sterols (i.e., sitosterol, stigmasterol) were also found. Lactose, a disaccharide, and cholesterol were identified in 4 samples, respectively (see Supplementary Data 1). In the animal calculi, in addition to fatty acids, ubiquitous terpene metabolites (i.e., limonene, eucalyptol, citronellol, and myrcenol) were traced.

### NGS analysis

Ancient DNA (aDNA) was successfully detected in five individuals: 65B+5B, 33IX, 102, 13+12C, and 39VB. All sequences obtained from NGS analysis are listed in Supplementary Data 2. The statistics about raw data, quality control paired-end reads, amplicon assembled, and alignment analysis are reported in Supplementary Data 3. To exclude any potential taxonomic misrepresentation associated with loss of AT-rich DNA fragments in ancient samples, following the guidelines of Dabney et al.^46^ and Mann et al.^47^, we also skimmed the GC content, measuring a %GC not significantly high (consistently <40). Ancient plant sequences are shown in Supplementary Table 6. Sample 13+12C revealed the presence of two clusters of amplicons which differ for a single nucleotide (A/G; evidenced in grey in Supplementary Table 6), most probably linked to a polymorphism of the original plant material. Thus, after the application of stringent authentication criteria, the taxonomic classification was done by Nucleotide Blast software. Details of BLAST matchings (i.e., scientific name of the species and relative accession number, and the best max score, query coverage, *E* value, and percentage identity) for the species identified as possible sources for aDNA were reported in Supplementary Data 4. In the samples 65B+5B, 33IX, and 13+12C we detected up 10,952, 132, and 7048 reads, respectively, all mapping to the *matK* barcode gene of the fig trees (*Ficus* sp. L.). In addition, 3046 reads relative to *matK* from different genera of Rosaceae family were found in calculus 102, while up to 114 reads mapping on the same chloroplast gene from several species belonging to Lamiaceae family in the sample 39VB.

## Discussion

Dental calculus, or tartar, is the result of bacterial plaque mineralisation adhered to the teeth surface. Its accumulation is progressive and even after fossilisation, it may preserve and protect from external damage biomolecules (e.g., DNA) and various microremains (e.g., phytoliths, starch, pollen, spores, plant tissues, sponge spicules, diatom frustules, inorganic soil particles), indicating what has been ingested/inhaled by an organism^2,4,48–52^ The precise timespan implicated in dental calculus growth is not yet understood; indeed, its formation and composition can be highly variable among individuals. For this reason, it is not possible to assess when, within the lifetime of a living being, a specific microparticle has been trapped in the mineral matrix^1,7,53,54^. More and more studies have been focused on the analysis of dental calculus, not only for human but also animal samples, where palynological assemblages are used for efficiently reconstructing feeding ecology and paleoenvironment^1,5–7,55,56^. In this study, dental calculus investigation yielded a great variety of microparticles, chemical markers, and aDNA sequences. The systematic contamination controls implemented in our work confirmed that the microdebris and aDNA detected from the Neolithic samples were original and not a result of laboratory environmental pollution.

In ancient dental calculus analysis, there are still many concerns about the pathways of inclusion for microdebris, especially for starch, the main nutritional component in the human diet. However, this type of microparticle may survive in niches protected on teeth surface and avoid oral digestion performed by alpha-amylase^57,58^. In the investigated series, two starch morphotypes were found, suggesting the use of wild grasses and cereals. Unfortunately, no Poaceae macrobotanical dataset supporting our results is available for Grotta Mora Cavorso. The archaeobotanical record from early Neolithic (6th millennium BCE) Italian sites provides evidence for the cultivation of various cereals. In general, *Triticum* was the best-represented genus^59–63^ and, for central Italy, barley and wheat were the first cultivated grains (e.g., La Marmotta site, Latium)^61^. Noteworthy is the recovery of Triticeae starches in Neolithic human tartar from Grotta Continenza (Fucino Basin, Abruzzo region)^3^, this providing further evidence to support our finding for the morphotype I. Particular attention must be paid to the discussion of starches named in this study as morphotype II (polyhedral grains), since often the isotopic signal relating to the consumption of C_4_ plants in the Mediterranean area seems to be masked^64^ or declared as unrealistic^65^. We were able to identify starches from Panicoideae subfamily. Thus, our results suggest that the individuals living in the surroundings of Grotta Mora Cavorso were already using wild C_4_ plants during the Neolithic, even for feeding animals. Published data on calculus have confirmed the presence of such a type of starch as early as the early Mesolithic in north and central Italy^3,66^. Seeds, starches, and phytoliths of millets have been found in several sites around the Mediterranean Sea^67–72^ and even in southern and northern Italy, although in some cases the identification remained uncertain^59,60^. *Panicum* and/or *Setaria* are possible genera to which the ancient starch granules recovered in our samples can be assigned, even though the so-called minor millets (i.e., *Echinochloa* sp. P. Beauv., *Brachiaria* sp. (Trin.) Griseb.) cannot be excluded, given the morphology of their starch granules^69^. No phytolith was recovered from calculi. This type of microfossils, in fact, would have helped us in distinguishing between the species of Paniceae^73^. Dental calculus analysis must be considered a qualitative study. Indeed, its multifactorial aetiology makes it impossible to define the quantity of ingested food, to distinguish between gender and age classes of individuals, and to understand when and how microparticles were trapped inside the mineral matrix. Therefore, we can evaluate only the food’s presence in the calculus, and not those absent. However, in this study, despite our having analysed many samples (a total of 55), no grains of starch from legumes were found. The scarcity of pulses in archaeological sites is a common feature in the whole north-western Mediterranean^71^, and the cultivation of legumes since the Early Neolithic^59^ and during CA of central Italy^74^ appears absent or uncertain. Even in ancient dental calculi, the lack (or scarcity^39^) of Fabaceae starch grains is quite frequent^3,66,75^. This phenomenon may be associated with the structure and physicochemical properties of legume starch, rich in resistant starch (about 17–50% of the dry matter), compared to that of cereals, roots, and tubers^76–79^. Thus, to date, this typology of starch has proved scarcely detectable in dental calculus. However, the possibility that legumes were consumed in limited quantities or only seasonally also needs to be considered.

Familiarity with plant foods was also confirmed through GC-MS analysis of human and animal dental calculi. Indeed, the evidence of terpenes, carotenoids, and phytosterols could testify to contact with plant material^43,45^. Terpenes and terpenoids, aspecific compounds with a volatile nature, coumarins, and tartaric acid are contained in fruits, leaves, and bark of a wide range of species^80–83^. Terpenes have been already detected in archaeological artefacts^44,45,84,85^. The presence of angustifoline in one sample is interesting. In literature, this alkaloid is mainly associated with *Lupinus* sp. Seeds and described as a minor component^86^, although quinolizidine alkaloids are also synthesised in several tissues of other Fabaceae genera, for instance *Genista*, *Cytisus*, and *Laburnum*^87,88^. If this molecule really derived from ingestion of lupin beans, the present outcome would constitute the first clue of consumption of legumes in central-southern Lazio during the Neolithic. Unfortunately, this hypothesis was unconfirmed either our own microscopy analysis on calculus (e.g., starch), or by carpological remains found at the site or in coeval contexts. Lactose, a sugar present only in milk and derivatives, was identified in the human calculi, corroborating palaeontological theories about the livestock of caprines by members of the Neolithic community who lived near Mora Cavorso to obtain dairy products^89^. Fatty acids are the main components of animal fats and plant oils. Unfortunately, it is difficult to associate them to specific sources, as they vary in quantity in the different foods and are not specific biomarkers^90^. Compared to investigations applied on archaeological organic residues^84,91,92^, in the case of dental calculus, linking a lipid profile to a single organic material is not straightforward. Indeed, degradation processes (e.g., cooking) may alter the number of carbons in the fatty acids, while dental calculus growth might affect the incorporation rate of these molecules in tartar, modifying their natural ratios^44,49,93^. For this reason, both plant and animal tissues could be defined as sources of the detected medium and long-chain fatty acids, but the odd ones could be also attributed to bacterial or ruminant lipids^44,91–94^. Although organic compounds are prone to oxidation^95^, archaeological ceramics and dental calculus have proved to be preservative for a wide range of molecules, including PUFAs^39,93,96,97^. The identification of omega-3 fatty acids in 16 Neolithic samples suggested intake of dried fruits (e.g., hazelnuts) and/or plant oils^98,99^, as supported by archaeobotanical records from the site^100^. Nevertheless, considering the proximity of the cave to Aniene River, consumption of aquatic organisms (e.g., freshwater fish) cannot be excluded due to their richness in PUFAs^101,102^.

Pollen is a powerful proxy in palaeoecological and biostratigraphic studies. Indeed, it has a high potential of preservation due to the major component of its tough outer layer (exine), the sporopollenin^103^. Only a few studies have looked at this type of palynomorphs in the human calculi^58,104–108^, although this dental deposit has turned out to be a preservative for highly degradable molecules, such as aDNA^4,14,18,109^. Dental calculus formation is fast^110^, making it possible to trace in tartar both morphologically unaltered and slightly degraded microparticles. The most common degradation events affecting these microdebris are oxidation *phenomena* of organic molecules, alteration by digestive enzymes (e.g., *alpha*-amylase), and oral microbiota activity^111^. However, these events precede the entrapment into calculus and/or occur on the outermost layer of dental plaque during its growth. Sporopollenin is a polymer that resists degradation by organic and inorganic chemicals. This raises the question: why should it remain unaltered during the formation of soil sediments^112^ and not within a matrix that calcifies in about two weeks? Sometimes, pollen grains from tartar seem dehydrated and/or altered. This could very likely be attributed to events prior to their inclusion. Instead, pollen with visible cytoplasm (or part of it) might be due to the rapid isolation process in the mineral matrix. In general, the pollen types retrieved from our dental calculi were found to be coherent among the samples, most likely because animals and humans inhabited the same areas around the cave. It is not possible to know if these palynomorphs are derived from food, natural substances (also used for medicinal purposes), or dirt/dust present on leaves or chewed foods (e.g., honey, resins, flowers/ inflorescences) but they certainly constitute evidence of plants. Concerning arboreal pollen types, a mountain taxon (i.e., *Fagus* sp. L.), evergreen trees (e.g., *Quercus*), and conifers were detected, followed by Fabaceae undifferentiated, whose *habitus* ranges from giant trees to small annual herbs. Among them, the palynomorphs characterised as Pinaceae, given their good state of preservation, were tentatively ascribed to the *Pinus* L. species. Cupressaceae pollen, instead, remained classified at family level, being considered morphologically uniform^32^. This palynomorph is generally scarce in ancient sediments and one of the most underrepresented in archaeological contexts. With respect to these findings, we know that Gymnosperm products (e.g., wood, resins, needles, nuts, and inner bark) have been widely used since prehistorical times^43,105^. Several archaeobotanical records have demonstrated the use of coniferous species in the Mediterranean basin^71^, especially in the Italian peninsula, since the Neolithic^113–115^. These pollen types suggested woodland patches in the surroundings of the cave, in accordance with palynological analysis at Grotta Mora Cavorso^116^, but also provided additional evidence for evergreen Mediterranean species, during the Neolithic period, in the Upper Aniene River Valley. The systematic determination of Poaceae, Cyperoideae, and Cheno-Amaranthaceae palynomorphs is challenging due to the uniformity in their pollen^35,117^. In addition, most of the Asteraceae subfamily Asteroideae pollen grains derived from herbs; thus, the specific attribution of this microfossils is difficult. Although a low taxonomic identification reduces palaeoecological conclusions, the evidence of these microgametophytes is usually interpreted as indicative of dry prairies and swamplands characterised by halophytes (salt-tolerant plants as Cheno-Amaranthaceae) and terrestrial and/or helophytic herbs (as Cyperaceae). This scenario suggested a warmer climate condition in the Upper Aniene River Valley and/or the possible frequentation of riparian forests/margins of river, also given the pollen uncertainly attributed to *Salix* genus, which includes deciduous trees and shrubs mainly distributed across moderate climate zones^34^. The detection of *Vitis* palynomorphs in human dental calculus is of particular interest because of the low presence in both pre-agricultural and post-agricultural pollen diagrams. The viticulture has been associated with classical civilisations or other socially structured societies^118^, although grapevine utilisation has been registered since prehistorical periods, that is before its domestication^119^. The cultivation of grapes, indeed, has played an important cultural and economic role throughout human history^120^. As Rottoli and Castiglioni^59^ stated, wild grape has been registered in Italy during the early Neolithic, but it did not seem to play a prominent role in the diet, as suggested by the limited number of macrobotanicals. Thus, the ancient pollen found in our sample, not attributable to wild or domestic forms, could represent proof for interaction/familiarity of the early Neolithic people with this plant species in central Italy, this alongside the possibility of chance inhalation. At palaeoecological level, this result lies within the general framework inferred by the other pollen types. For example, the wild grapevine is a heliophilous plant, usually growing along riverbanks and in alluvial/colluvial deciduous/semi-deciduous forests around the Mediterranean Basin^71,121,122^. Non-diagnostic fibres of plant origin and spiral vessels were recovered in all samples. These remains were particularly abundant and could suggest chewing of raw plant material (both for humans and animals) and/or an extra-masticatory use of teeth^52,123^.

Some microparticles found in the ancient calculi (e.g., trichomes, fern spore, fragment of pappus, and softwood) can be considered as potential sources of information regarding past natural environments and/or inhabited areas^52^. They could have been inhaled or accidentally ingested, even as dirt on food. The fragment of coniferous wood in the tartar of pine marten (*Martes* sp.) is not so unexpected, as this mustelid is known to prefer coniferous forests as an ecological niche^124^. Therefore, this microdebris could have been inhaled by the animal during its movements on the trees. Another noteworthy category of microparticles was that of difficult taxonomic placement (i.e., unknown origin), which might potentially shed light on water sources, environments (e.g., riparian, lacustrine), and climate. Three different classes of organisms may have contributed to the production of this type of NPPs: heliozoan protists, dinoflagellates, and sponges. Due to the treatment used for dissolving dental calculus, a calcareous nature for these microremains should be excluded, while in all probability a silica origin can be assumed. Morphology and size of these spherical particles perfectly fit with centroheliozoa and dinoflagellate cysts, predominantly living in freshwater continental habitats^125–128^. Centrohelids, organisms belonging to Choanocystidae and Raphidiophryidae families, exhibit distinct outer radial silica scales; among them, *Choanocystis aculeata* Hertwig & Lesser (basionym: *Acanthocystis aculeata*) shows the same appearance and dimension range as the ancient debris^129,130^. Dinocysts, instead, can sometimes possess spines on their superficial ornamentation. This resistant resting stage is unique among protists and most frequently precedes a period of adverse growth conditions (e.g., winter season). As palaeoecological *proxies* of aquatic environment, they can also indicate distance from the coastline, productivity, surface temperature, and surface salinity. Dinocysts are deposited in the water-sediment interface, accumulate in the sediments, and remain dormant until the growth season^127^. Thus, heliozoans are found mainly on/near the benthos^131^, while the dinoflagellate cysts behave like a sediment particle^127^. Unfortunately, a specific taxonomic identification was not possible, but both the organisms live near the bottom of a basin (possibly freshwater sources, given the possible areas of frequentation of the individuals buried at Mora Cavorso). Therefore, questions that remain unanswered are: how were these microdebris trapped in dental calculus of so many human individuals? Were they all contained in the same water source? Was perhaps some sediment mistakenly collected along with the water? Did people feed on benthic organisms (e.g., amphipods, shellfish)? To conclude, although displaying highly variable body shape and size^132^, freshwater sponge gemmules (Demospongiae, order Spongillida) have usually dimensions three times larger than our ancient microremains; thus, even if very similar in morphology, the resting bodies of sponges can be excluded with a high level of confidence.

To specifically detect traces of aDNA deriving from plants potentially ingested by the early “Neolithics”, we amplified *matK* chloroplast barcode gene in calculus samples and then sequenced it by NGS approach. It now remains to investigate in parallel also other plastid and/or nuclear barcode genes, for obtaining a higher variability of conserved sequences. However, the mutation rate of plastid genome is higher than nuclear and mitochondrial one, making plastid genes more discriminating than the others (e.g., Internal Transcribed Spacer ITS). It is necessary to bear in mind that aDNA is prone to damage and degradation events and only a few very short fragments can survive and this study should be considered as preliminary methodological research focused on plant DNA analysis from ancient dental calculus. Thus, the proposed method was created and applied taking into account the following considerations: (i) *matK* barcode gene is present in high copy number and the relative sequence is expected to be easily amplifiable, being located on chloroplast genome; (ii) amplicon for *matK* gene used in this study is 217-bp long, making it adequate for aDNA analysis; (iii) sequencing may introduce biases due to the fragmented nature of aDNA; (iv) aDNA damage (e.g., depurination, conversion of cytosine to uracil, increment of cytosine to thymine changes) is generally consistent and often difficult to assess, highlighting the importance of sequence authentication; (v) the limitations associated with the use of modern reference sequences must be evaluated. Given this background, we were able to authenticate the presence of aDNA from different plant genera. Taking for granted basic recommendations, such as field/storage contamination, parallel processing of negative controls, laboratory procedures and database suitable for aDNA analyses, and taxonomic resolution^16^, we detected 9785 authentic reads corresponding to three plant sequences in five tartar samples out of nine. In detail, we found only one type of sequence per sample (except for 13+12C, which presented two clusters of amplicons belonging to the same species). Our data suggested that three Neolithic individuals could have ingested portions of *Ficus* sp. Excluding accessions with origins far from the Mediterranean area (e.g., *F. adhatodifolia* Schott, *F. hirta* Vahl.), *F. carica* L. may be the species that left its DNA fragments in the dental calculi. The recovery of figs in Neolithic archaeological contexts is quite rare. In Italy, pollen and macro-remains of this sub-mediterranean species, living mainly on dry and sunny soils, have been found in central and northern regions (i.e., Lazio, Umbria, and Friuli), Sicily, and Sardinia^59,63,133–136^. The other two specimens, instead, showed possible use of Rosaceae (sample 102) and Lamiaceae (sample 39VB). BLAST matching revealed several species for the sequence retrieved in sample 102. However, we filtered this list by eliminating non-native species for the Mediterranean area. Indeed, as we are referring to prehistoric samples, it would not make sense. Among the plants belonging to *Pyrus* L. and *Sorbus* L. genera (see Supplementary Data 4), only *P. communis* L. and *S. aucuparia* L. can be considered species used for dietary purposes by the individual, both of them being native to Europe and western Asia. In fact, beyond figs, other edible wild fruits (including *Pyrus*) could have been gathered by the first sedentary communities of farmers, playing a key role in their subsistence patterns, as attested in North of Italy^59^. Sample 39VB revealed a *matK* sequence attributable to the following *taxa*: *Saccocalyx saturejoides* Coss. & Durieu, *Argantoniella salzmannii* G.López & R.Morales (synonym of *Satureja salzmannii* (Kuntze) P.W.Ball), six species of *Thymus* L. and six of *Origanum* L. The first two species should be deleted since they are non-endemic of the Mediterranean. Among the thymes, *T. pulegioides* L. and *T. vulgaris* L., both native to southern Europe, are small spreading subshrubs with strongly aromatic leaves which may have been employed by the individual for dietary/medicinal purposes. Regarding oregano, all accessions were plausible, as these perennial herbs are native to the Mediterranean region. A few records of Lamiaceae around the Mediterranean and in central Europe during the Neolithic period have been reported in literature^137–139^. In support of the possible use of Lamiaceae in the diet of the studied Neolithic individuals, it should be remembered that biochemical markers, such as monoterpenes and monoterpenoids, tetraterpenes, sesquiterpene derivatives and sesquiterpenoids, were also found in some human dental calculi in this study. The presence of plant aDNA from calculus testifies the consumption of fruit and aromatic species as foods, surely complementing the diet with vitamins and minerals. Unfortunately, no macrobotanicals have been documented for these species at Grotta Mora Cavorso. However, the multiple validation criteria which we applied, corroborated by the macrofossil evidence well-documented throughout the Italian Neolithic, indicate that the detected biomolecular record is authentic.

In conclusion, little is known about the role of wild plant foods and how they contributed to the diet of the earlier inhabitants of our Peninsula. Thus, in this contribution, we present the whole archaeobotanical assemblage preserved in the dental calculus of early Neolithic people, probably belonging to different communities which could have exploited Grotta Mora Cavorso as a funerary shelter, to infer food preferences and habitat. Due to the limited amount of tartar left on the teeth of the analysed specimens, we can only speculate about the plant species’ value in the diet. Our results should not be considered merely as evidence of regular dietary use but they reveal the presence and the prehistoric knowledge of various plants. The mineralised dental biofilm has returned and preserved microdebris, aDNA, and biomolecular markers. The evidence of starch from Panicoideae grasses was of peculiar interest. Indeed, our research suggests the consumption of their caryopses by the Neolithic people in central Italy, together with a mixture of other edible species (e.g., grains, figs, Rosaceae, aromatic herbs). The palynological record retrieved from the human samples, as well as NPPs, outlines a general vegetational framework near the cave characterised by deciduous/semi-deciduous forests. Pollen from animal tartar also confirms such a scenario. This specific paleoenvironment could have offered a favourable location for the development of semi-permanent settlements where a subsistence economy was growing, most probably directed towards the exploitation of available natural plant resources.

## Methods

### Archaeological context

In 2001, the speleological group *Shaka Zulu* of Subiaco has discovered a multi-tunnel karst cave, Grotta Mora Cavorso, located in South-Eastern Latium (Upper Aniene River Valley, inner Apennines, central Italy) (Fig. 1a). The complex and rich stratigraphy spans from the Upper Palaeolithic to the modern age and contains one of the largest burial deposits in Italy and in the Mediterranean area dated the early Neolithic. Ten years of multidisciplinary investigation at the site have provided evidence about this peculiar anthropized cave, with a specific focus on Neolithic occupation^89,100^. Indeed, the recovery of human and animal bones from the inner rooms (Upper and Lower Room, UR and LR; Fig. 1b) has encouraged systematic archaeological excavations by the University of Rome Tor Vergata. From the Neolithic layers (Supplementary Fig. 1), mainly attributed to an Early phase, skeletal remains of humans have been extracted. Three individuals found in the Upper room were still in partial anatomical connection, while around 600 bones, chaotically piled and belonging to at least 28 individuals, have been dug up from the Lower Room. The preliminary studies on aDNA have suggested an indigenous genetic ancestry, partly from the Near East, for the community members of Grotta Mora Cavorso^89^. Investigations on carbon and nitrogen isotopes have indicated a diet mainly based on meat^64^. The faunal assemblage from the Neolithic layers of the cave (Fig. 1, Soundings B1-C-D) consisted in domestic and wild mammals (among them: *Ovis* vel *Capra*, *Cervus elaphus* L., *Vulpes* sp., *Martes* sp., *Lepus* sp., *Bos taurus* L., and *Sus* sp.). Paleo-dietary evidence and palaeontological record allowed us to hypothesise a subsistence strategy of the Neolithic community oriented towards stock-farming activities. Indeed, domesticated animals could have been bred for producing dairy products (e.g., caprines) and meat (e.g., pigs). In parallel, the occurrence of red deer and wild boar bones, often showing butchery marks due to occasional hunting activity, and of the smaller mammals (including carnivores) would indicate the existence of wet woodlands during the Neolithic^81,131^.

### Sampling and decontamination protocol

The palaeontological and anthropological specimens investigated in this work belong to a collection hosted at the Department of History, Culture and Society (University of Rome “Tor Vergata”, Italy), under the supervision of Prof. Mario Federico Rolfo. In October 2019, before any sampling, we identified and documented suitable supragingival dental calculus deposits (Fig. 1c). Fifty-five tartar samples were analysed: 11 of them were collected from the human skeletal remains identified as individuals (i.e., teeth localised in mandibular or maxillary bones), 33 were sampled from human isolated teeth, and 11 were obtained from animals (see Supplementary Table 1). All human and animal samples were undergone to a multi-step extraction method for the analysis of molecular markers (GC-MS) and microparticles (OM). In addition, about 25–30 mg of calculus from nine human individuals (see Supplementary Table 1) were subjected to aDNA extraction and NGS. Following a regime of intensive laboratory cleaning^39,140^, validated by lab contamination tests (Supplementary Table 5), the sampling of calculus was done in the cleanroom facility of the Department of Biology of the University of Rome Tor Vergata, removing it from the tooth enamel by an autoclaved dental pick. To prevent any contamination, during the processing, the labs were used exclusively for dental calculus, no other material was processed, and all the steps were conducted under a sterile vertical laminar flow hood. Considering all recommendations reported in Mercader et al.^141^, Mann et al.^16^, and Farrer et al.^13^, calculus decontamination protocol was performed with ultraviolet radiation (UV) and 5% sodium hypochlorite (NaClO) immersion treatments, to minimise the exogenous content of the outer surface of ancient calculus flakes. Preceding the decontamination protocol, 15 human and 3 animal randomly selected calculi were washed by sterile water, which was examined by OM, to confirm the efficacy of the method. No microparticles was detected after sterilisation.

### Chemical markers and microdebris analysis

After the decontamination procedures, the mineralised plaque retrieved from different teeth was combined into a single tube where solubilisation treatment (3% HCl, 2 h) and preparation of the samples (resuspension in hexane, 2 h) was performed according to D’Agostino et al.^39^ The sample fraction subjected to GC-MS technique was analysed, in triplicate, using a QP2010 system (Shimadzu, Kyoto, Japan) and helium as gas carrier (constant flow: 1 mL/min). Two microliters of extract were loaded into the instrument set at 280 °C (splitless modality). The temperature gradient consisted in an initial step at 60 °C for 5 min, to reach then 150 °C for 5 min, 250 °C for 5 min, and 330 °C for 25 min, at a rate of 6 °C/min. The electron impact was configured at 70 eV (scanning from 100 to 700 m/z), while the ion source temperature at 230 °C, the interface temperature at 320 °C, and the solvent cut time at 6 min. Mass spectra of the detected analytes were identified using the NIST (National Institute of Standards and Technology) Library 14 database. Literature data and scientific food databases were also consulted for reconstructing food categories and plant species.

The pellet deriving from the decalcification step was employed to evaluate the presence of microparticles by OM (ZEISS Axio Observer 7 equipped with polarised filters and Zen imaging software 2.6, operating at different magnifications). Starch granules, palynomorphs, NPPs, and palynodebris elements were identified on morphometric features, grouped into preliminary types based on a shared morphology, and described using conventional nomenclature^23,142^. Each microparticle was measured in triplicate. A dimensional range was reported for those belonging to the same taxon. Literature works, our reference collections and databases of wild and domestic plant species assisted and made the identification possible.

### Experimental reference collection for microscopy

A reference collection (Supplementary Fig. 2) composed of plant microremains extracted from fruit fragment of modern Asteroideae (Cass.) Lindley and Cichorioideae (Juss.) Chevall. subfamilies were specifically created in a separate laboratory. Capillary bristles from several pappi of *Erigeron* sp. L., *Hieracium* sp. L., *Sonchus* sp. L., and *Taraxacum* sp. F. H. Wigg. were collected with a drop of ultrapure water, mounted on microscopic slides, and observed at optical microscope.

### DNA extraction, NGS analysis, and alignment to reference sequences

A sufficient amount of calculus for aDNA analysis was available only for nine human samples (Supplementary Table 1). The first steps of the investigation (e.g., aDNA extraction) were conducted in dedicated facilities of the Departmental Centre for Ancient DNA Studies (Villa Mondragone, Rome, Italy), specifically designed to eliminate potential contaminations, and in the Laboratories of Archaeobotany of the University of Rome Tor Vergata DAPHNE (Diet, Ancient DNA, Plant-Human Nexus, and Environment), following standard precautions and recommendations^16,143–146^. Amplicon sequencing and bioinformatics analyses, instead, were carried out by Bio-Fab research S.r.l (Rome, Italy). Following decontamination, each sample was incubated with 1 mL of lysis buffer (EDTA 0.5 M pH 8; 0.25 mg/mL Proteinase K; 0.05% Tween 20) for 48 h at 37 °C in agitation, according to recommendations reported in Le Moyne and Crowther^147^. After that, NucleoSpin™ Plant II kit (Macherey-Nagel) was used to isolate and purify genomic DNA from the supernatant. aDNA was treated with USER Enzyme (BioLabs, New England) and Fast DNA End Repair Kit (Thermo Fischer Scientific), according to the manufacturer’s guidelines. Once purified by NucleoSpin Gel and PCR Clean-up (Macherey-Nagel). The pellet was incubated in 0.2 M hydrochloric acid (HCl) for 6 h and observed by OM for microparticles, as reported above. A fundamental step consisted in a PCR enrichment of the samples by amplifying maturase K (*matK*) barcode gene (present in the chloroplast genome), to exclude bacterial (dominant in calculus) and animal DNA (deriving from food). *MatK* primers were those reported in Gismondi et al.^100^ (F1 and R2), adequately ligated to adapters for NGS library preparation (TCGTCGGCAGCGTCAGATGTGTATAAGAGACA-*Primer F1*; GTCTCGTGGGCTCGGAGATGTGTATAAGAGACAG-*Primer R2*). Each PCR reaction contained: aDNA extract (10 ng), 0.3 μL of Accu Taq LA DNA Polymerase (5 U/μL, Merck), 5 μL of 10× reaction buffer, 1.5 μL MgCl_2_ (25 mM), 1 μL dNTPs (10 mM each NTP), and 1 μL of each primers (10 pmol/μL), for a total volume of 50 μL. PCR thermal cycling conditions were as follows: initialisation (95 °C, 5 min); 60 cycles of denaturing (95 °C, 30 s), annealing (56.4 °C, 20 s), and elongation (72 °C, 20 s); final elongation (72 °C, 10 min); hold step (4 °C). Negative controls were included in the PCR analysis. The quality of the amplicons was monitored by visualising on 1.5% agarose gel. Only five samples resulted suitable for continuing the process. NGS protocol started from DNA library preparation, using Nextera XT DNA Library Prep kit (Illumina, San Diego, CA, USA). The library was quantified by a Qubit 2.0 Fluorometer (Invitrogen) and validated by Bioanalyzer 2100 (Agilent Technologies, Palo Alto, CA, USA). DNA sequencing was completed using the MiSeq instrument (Illumina, San Diego, CA, USA), with a 2 × 300 paired-end run and double indexing of the library (acceptance criteria: kit chemistry v3; 600 cycles; acceptability range (%): ≥70; results (%): 78.14). A final sequencing depth between 423,000 and 143,000 reads per sample was obtained. One blank negative control (water) was processed in parallel with the calculus samples, both in extraction and library construction steps, for the proper authentication of aDNA. A quality control paired-end reads and an estimate of the assembled amplicons were conducted. Alignment analysis was done for taxonomic classification by Nucleotide Blast software, using NCBI Reference Sequence (RefSeq) database (https://www.ncbi.nlm.nih.gov/refseq/). The aligned amplicons were then clustered for similarity. Several bioinformatics tools were applied to perform the quality control of raw data (FastQC v0.11.9; cutadapt v3.4), alignment (BBMap v38.79; Bowtie2 v2.3.4.3 by Ben Langmead/Freebayes v1.0.0; bedtools v2.29.2), and variant calling (samtools/bcftools v1.9 using htslib 1.9). MapDamage and snpAD (v0.3.5d) were used to check the authenticity of the ancient sequences and to implement an iterative method for evaluating error rates, and genotypes frequencies from the data. These estimates were used in a second step to calculate the posterior probabilities for each genotype and produce a Variant Call Format. Thus, aDNA consensus sequences were generated.

### Reporting summary

Further information on research design is available in the Nature Portfolio Reporting Summary linked to this article.

## Supplementary information

Supplementary Information

Description of Additional Supplementary Files

Supplementary Data 1

Supplementary Data 2

Supplementary Data 3

Supplementary Data 4

Reporting summary

## Data Availability

Data relative to the present research have been provided in the text and in Supplementary Information and Supplementary Data.
