# Supplementary material for: Neolithic dental calculi provide evidence for environmental proxies and consumption of wild edible fruits and herbs in central Apennines
Source: Commun Biol. 2022 Dec 19;5:1384. doi: 10.1038/s42003-022-04354-0 (PMC9763411; doi:10.1038/s42003-022-04354-0)
Supplement: Supplementary file 1 — Supplementary Information [file 42003_2022_4354_MOESM1_ESM.pdf]

SUPPLEMENTARY FIGURE 1. Radiocarbon dating. Table of measurements obtained by OxCal[1](version 4.4.4), using the IntCal20 curve[2], and relative details and graphs.

Laboratory	Material dated (sample type)	Radiocarbon date BP	Calibrated date BCE
Lecce LTL6124A	B1/d8/US300 (charcoal)	6505±50	5561-5365
Lyon-3504	SI US1 (human bone)	6405±35	5474 - 5314
Lyon-5202	SS US (charcoal)	6275±45	5333-5205
Lecce LTL6123A	C/A2 US22 (charcoal)	6000±45	5008 - 4783

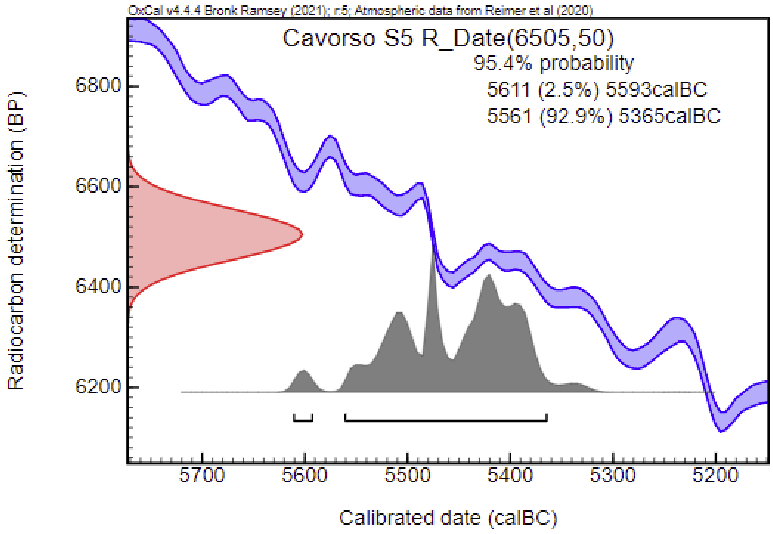

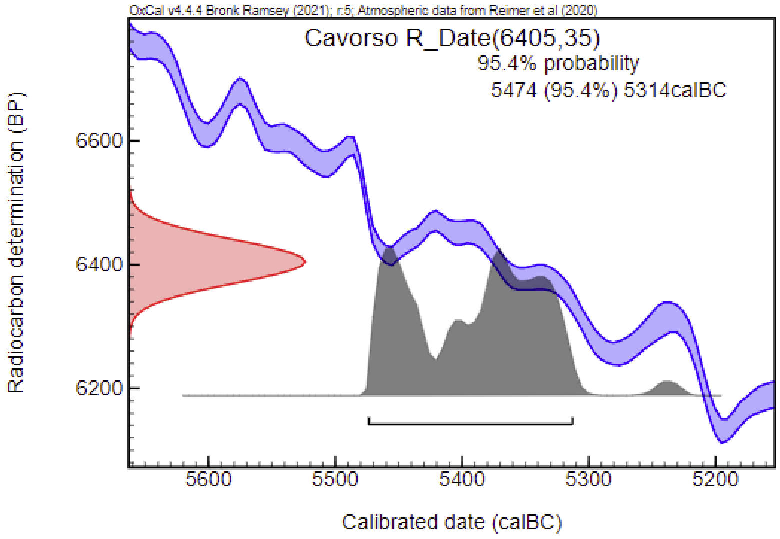

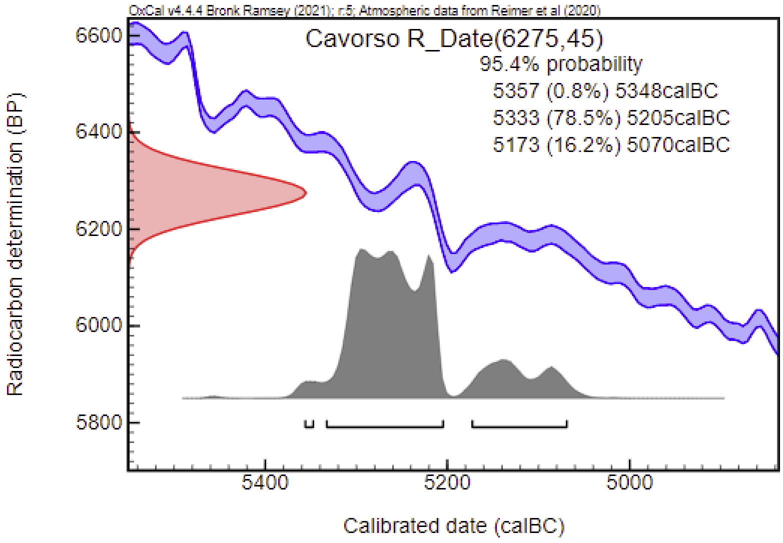

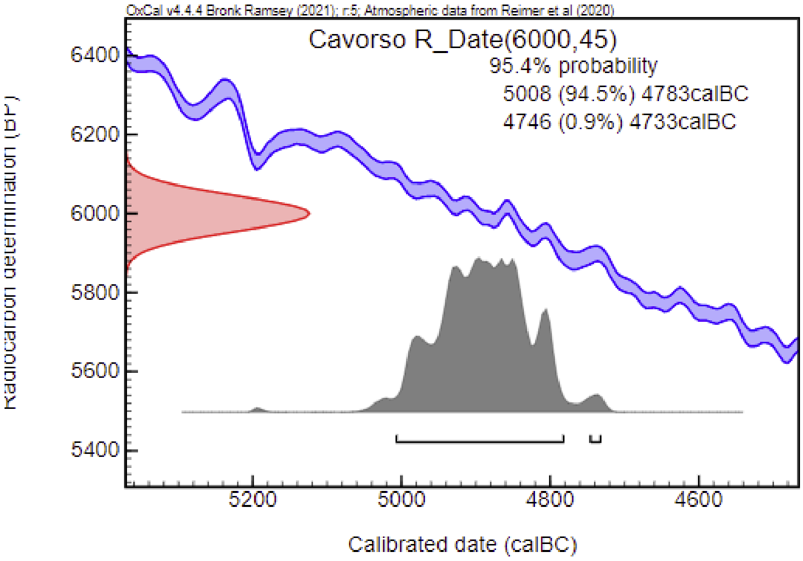

Supplementary References

1. Bronk Ramsey, C., OxCal 4.4.4. Available from: http://c14.arch.ox.ac.uk/oxcal. (2021).
2. Reimer, P. J. et al. The IntCal20 Northern Hemisphere radiocarbon age calibration curve (0–55 cal kBP). Radiocarbon 62, 725-757 (2020).

Supplementary Figure 2. Experimental reference collection.

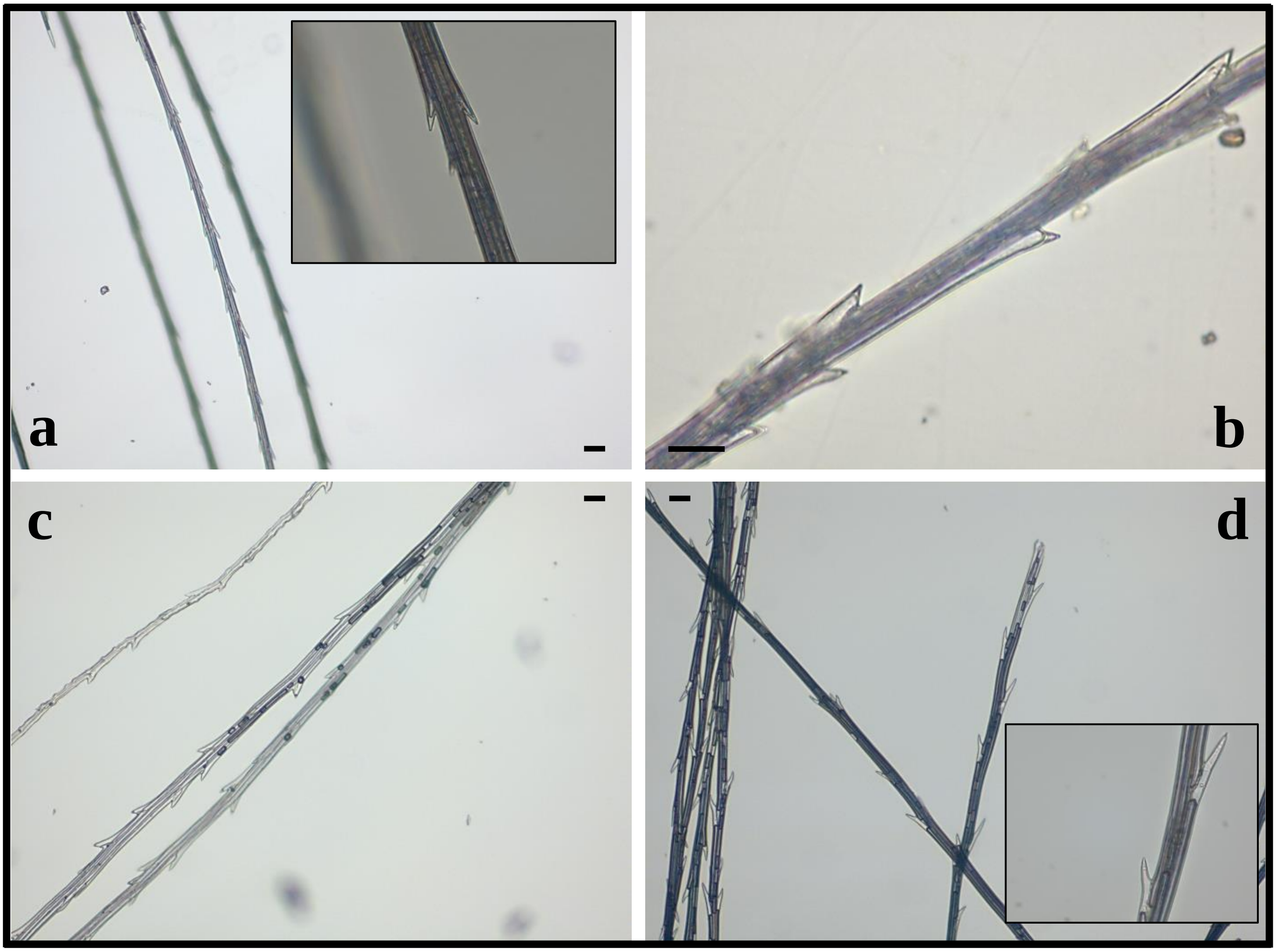

Representative images from modern plant materials observed at light microscope were shown. Fruit fragments of: **a** *Erigeron* sp., **b** *Hieracium* sp., **c** *Sonchus* sp., and **d** *Taraxacum* sp. The scale bar indicates 20 µm.

SUPPLEMENTARY TABLE 1. Dental calculus samples and applied methods. Codes for human and animal samples subjected to Optic Microscopy (OM), Gas Chromatography-Mass Spectrometry (GC-MS), and Next Generation Sequencing (NGS). ND: not determined sex.

	Code	Sex/Age at death	OM	GC-MS	NGS
Human dental calculus samples (individuals)	**12E**	ND/Adult	x	x	
	**94A+127A**	ND/Adult	x	x	x
	**SS11**	ND/Adult	x	x	
	**102**	ND/Adult	x	x	x
	**33IX**	ND/Adult	x	x	x
	**SS44A+44B**	ND/Adult	x	x	x
	**39VB**	ND/Adult	x	x	x
	**13+12C**	ND/Adult	x	x	x
	**105B+105C**	ND/Adult	x	x	x
	**76B**	ND/Adult	x	x	x
	**65B+5B**	ND/Adult	x	x	x
Human dental calculus samples (isolated teeth)	**4star**		x	x	
	**76E**		x	x	
	**76H**		x	x	
	**138E**		x	x	
	**248**		x	x	
	**18star**		x	x	
	**138B**		x	x	
	**138F**		x	x	
	**12star**		x	x	
	**138A**		x	x	
	**74**		x	x	
	**240**		x	x	
	**13star**		x	x	
	**61star**		x	x	
	**11star**		x	x	
	**125A**		x	x	
	**126B**		x	x	
	**126C**		x	x	
	**146L**		x	x	
	**239**		x	x	
	**76I**		x	x	
	**234**		x	x	
	**236**		x	x	
	**13+12-23**		x	x	
	**11-12**		x	x	
	**76F**		x	x	
	**243**		x	x	
	**146H**		x	x	
	**76A**		x	x	
	**144A**		x	x	
	**238**		x	x	
	**12**		x	x	
	**251**		x	x	
Animal dental calculus samples	**A1**		x	x	
	**A2**		x	x	
	**A3**		x	x	
	**A4**		x	x	
	**A5**		x	x	
	**A6**		x	x	
	**A7**		x	x	
	**A8**		x	x	
	**A9**		x	x	
	**A10**		x	x	
	**A10(2)**		x	x	

SUPPLEMENTARY TABLE 2. Microdebris recovered from human dental calculus samples of Grotta Mora Cavorso. Amount and proposed identification of microparticles detected by light microscopy in calculi from human individuals. Starch Morphotype II: Panicoideae. Legend for pollen type: Fb, Fabaceae undifferentiated; GC, Gymnosperm Cupressaceae; Q, *Quercus* deciduous undifferentiated; S, *Salix*; ND, not determined pollen. Legend for the other microparticles: FF, Cichorieae/Astereae fruit fragment; Uo, Unknown origin microparticles.

**Code**	**Starch Morphotype II**	**Not determined starch**	**Total starches per sample**	**Single pollen grain**	**Fragment of plant tissue**	**Plant fibres**	**Other micro-remains**
**12E**						8	
**94A+127A**		2	2		1	10	
**SS11**						6	
**102**						14	
**33IX**		1	1	1 Fb, 1 GC, 2 ND		11	1 FF
**SS44A+44B**	15		15	1 Q		11	1 Uo
**39VB**						5	
**13+12C**				1 S		14	1 Uo
**105B+105C**						10	
**76B**						9	
**65B+5B**		1	1			13	
**Total**	**15**	**4**	**19**	**6**	**1**	**111**	**3**

SUPPLEMENTARY TABLE 3. Microdebris recovered from human isolated calculus samples of Grotta Mora Cavorso. Amount and proposed identification of microparticles detected by light microscopy in calculi from human isolated teeth. Starch Morphotypes: I, Triticeae; II, Panicoideae. Legend for pollen type: C/A, Cheno-Amaranthaceae; Cy, Cyperaceae; F, Fagaceae, *Fagus*; GP, Gymosperm Pinaceae; P, Poaceae; Q, *Quercus* deciduous undifferentiated; V, *Vitis*; ND, not determined. Legend for the other microparticles: T, stellate trichome; Uo, microparticles with unknown origin.

Code	Starch Morphotype		Not determined starch	Total starches per sample	Single pollen grain	Trichome	Plant fibres	Other micro-remains
	I	II						
**4star**	1			**1**			4	
**76E**								
**76H**							10	
**138E**					1 Q			
**248**		1		**1**			10	
**18star**					1 GP			
**138B**	5	1	1	**7**				
**138F**							5	
**12star**			1	**1**				
**138A**					1 C/A	1T	20	1 Uo
**74**		1		**1**				
**240**								
**13star**							11	
**61star**					1 Q, 1 Cy, 1 P		19	3 Uo
**11star**					1 ND	1 T	25	
**125A**		1		**1**	1 V			1 Uo
**126B**	1			**1**	2 Cy		15	1 Uo
**126C**							6	
**146L**							10	
**239**								
**76I**	1			**1**			9	
**234**	1			**1**			12	
**236**	2			**2**			14	
**13+12-23**					1 GP, 1 F		19	1 Uo
**11-12**							12	
**76F**							10	4 Uo
**243**							13	1 Uo
**146H**								
**76A**	1			**1**			12	
**144A**							2	
**238**							15	
**12**		1		**1**	1 P			
**251**					1 GP			
**Total**	**12**	**5**	**2**	**19**	**14**	**2**	**253**	**12**

SUPPLEMENTARY TABLE 4. Microdebris recovered from animal dental calculus samples of Grotta Mora Cavorso. Amount and proposed identification of microparticles detected by light microscopy in calculi from animal specimens. Starch Morphotype I: Triticeae. Legend for pollen: A, Asteroideae undifferentiated; Fagaceae, *Fagus*; Fb, Fabaceae undifferentiated; GP, Gymnosperm Pinaceae. Legend for the other microparticles: T, stellate trichome; TF, tracheid fragment from conifer wood with torus-margo pit; SV, spiral vessels; MS monolete spore.

Lab code	Paleontological code	Species	Starch Morphotype I	Not determined starch	Total starches per sample	Single pollen grain	Plant fibres	Other micro-remains
**A1**	**B1755**	***Bos taurus***						
**A2**	**B429**	***Ovis* vel *Capra***					9	1 MS
**A3**	**B2298**	***Cervus elaphus***				1 GP		
**A4**	**2927**	***Vulpes* sp.**						
**A5**	**1206**	***Martes* sp.**		1	1	1 Fb	10	1 T, 1 TF
**A6**	**C186**	***Sus* sp.**					10	
**A7**	**B1574**	***Ovis* vel *Capra***						
**A8**	**B2230**	***Ovis* vel *Capra***				1 F	16	
**A9**	**B544**	***Lepus* sp.**						
**A10**	**B1612**	***Cervus* sp.**	5		5	2 GP, 1 A		
**A10(2)**	**B1612(2)**	***Cervus* sp.**	1		1		11	3 SV
**Total**			**6**	**1**	**7**	**5**	**56**	**6**

SUPPLEMENTARY TABLE 5. Results of lab contamination tests by horizontal slide trap (others: fibres, hairs, dust residues).

Context	Location of traps (number)	Starches	Pollen grains	Others	Total
**Workday**	workbench (15)	2	3	2	**7**
	floor (12)	6	8	3	**17**
	shelf (8)	2	1	0	**3**
	hood (6)	0	0	0	**0**
**After decontamination**	workbench (15)	0	0	0	**0**
	floor (12)	1	1	0	**2**
	shelf (8)	0	0	0	**0**
	hood (6)	0	0	0	**0**

SUPPLEMENTARY TABLE 6. Plant ancient sequences detected in dental calculi. aDNA sequences were shown for each sample. The single-nucleotide polymorphism detected in sample 13+12C between the two clusters of amplicons was evidenced in grey.

Samples	Cluster	Ancient sequence (5'-3')
**65B+5B**		TTCTAGCACAAGAAAGTCGAAGTATATATTTTACTCGATACAAACTATTTTTTTTTGAGGATCCACTGTAATAATGAAAA<br>AGATTTCTGCATATATACGCAAATCGGTCGATAATATCAAAATCCGATGAATCAGCCCAGGTCGACTTACTAACGGGATG<br>TCCTAATACGTTACAAAATCGCATTTTAGCCAACGATCCAATCAGAGGAATAATT
**33IX**		TTCTAGCACAAGAAAGTCGAAGTATATATTTTACTCGATACAAACTATTTTTTTTTGAGGATCCACTGTAATAATGAAAA<br>AGATTTCTGCATATATACGCAAATCGGTCGATAATATCAAAATCCGATGAATCAGCCCAGGTCGACTTACTAACGGGATG<br>TCCTAATACGTTACAAAATCGCATTTTAGCCAACGATCCAATCAGAGGAATAATT
**102**		TTCTAGCACAAGAAAGTCGAAGTATATATTTTATTCGATACAAACTCTTTTTCTTGAGGATCCACTGTAATAATGAGAAA<br>GATTTCTGCATATATGCACAAATCGGTCGATAATATCAAAATCCGACGAATCTGCCCAGGTGGACTTACTAATAGGATGC<br>CCCAATGCGTTACAAAATTTCACTTTAGCCAATGATCCAATCAGAGGAATAATT
**13+12C**	1	TTCTAGCACAAGAAAGTCGAAGTATATATTTTACTCGATACAAACTATTTTTTTTTGAGGATCCACTGTAATAATGAAAA<br>AGATTTCTGCATATATACGCAAATCGGTCGATAATATCAAAATCCGATGAATCAGCCCAGGTCGACTTACTAACGGGAT<br>GTCCTAATACGTTACAAAATCGCATTTTAGCCAACGATCCAATCAGAGGAATAATT
	2	TTCTAGCACAAGAAAGTCGAAGTATATATTTTACTCGATACAAACTATTTTTTTTTGAGGATCCACTGTAATAATGAAAG<br>AGATTTCTGCATATATACGCAAATCGGTCGATAATATCAAAATCCGATGAATCAGCCCAGGTCGACTTACTAACGGGAT<br>GTCCTAATACGTTACAAAATCGCATTTTAGCCAACGATCCAATCAGAGGAATAATT
**39VB**		TTCTAGCACAAGAAAGTCGAAGTATATACTTTATTCAATACAAACTCCTTTTTCTGCAAGATCCGCTATGATGATGAGAAA<br>TATTTCTGCATATACGCAAAAATCGGTCAATAATATTAGAATCTGATAACTCAGCCCGAATGGGCTTACTAATAGGATGC<br>CCAATGCGTTACAAAATATCACTTTAGCCAATTCTCCAATCAGAGGAATAATT
