# Supplementary material for: Neolithic dental calculi provide evidence for environmental proxies and consumption of wild edible fruits and herbs in central Apennines
Source: Commun Biol. 2022 Dec 19;5:1384. doi: 10.1038/s42003-022-04354-0 (PMC9763411; doi:10.1038/s42003-022-04354-0)
Supplement: Supplementary file 2 — Description of Additional Supplementary Files [file 42003_2022_4354_MOESM2_ESM.pdf]

Description of Additional Supplementary Files

SUPPLEMENTARY DATA 1: Chemical markers identified by GC-MS in dental calculus samples. Chemical compounds detected for each individual (except the samples A2, A3, A4, A7, and A9, which did not show significant molecules) were listed alphabetically, excluding n-alkenes and nalkanes.

SUPPLEMENTARY DATA 2: Amplicon assembled sequences. All sequences obtained from NGS analysis for each sample were reported.

SUPPLEMENTARY DATA 3: Bioinformatics analysis of NGS data. Statistics obtained from raw data (using FastQC v0.11.9), quality control paired end reads, amplicon assembled, and alignment analysis (Nucleotide BLAST) were reported in detail.

SUPPLEMENTARY DATA 4: BLAST matching. Scientific name (species), max score, query coverage, E-value, percentage (%) identity and accession number (GenBank code) of the nucleotide successions that highly matched with respect to matK ancient sequences.
