# Supplementary material for: Neolithic dental calculi provide evidence for environmental proxies and consumption of wild edible fruits and herbs in central Apennines
Source: Commun Biol. 2022 Dec 19;5:1384. doi: 10.1038/s42003-022-04354-0 (PMC9763411; doi:10.1038/s42003-022-04354-0)
Supplement: Supplementary file 7 — Reporting summary [file 42003_2022_4354_MOESM7_ESM.pdf]

nature portfolio

Corresponding author(s): Prof. Angelo Gismondi

Last updated by author(s): 23/11/2022

Reporting Summary

Nature Portfolio wishes to improve the reproducibility of the work that we publish. This form provides structure for consistency and transparency in reporting. For further information on Nature Portfolio policies, see our Editorial Policies and the Editorial Policy Checklist.

Statistics

For all statistical analyses, confirm that the following items are present in the figure legend, table legend, main text, or Methods section.

n/a	Confirmed	
☒	☐	The exact sample size (*n*) for each experimental group/condition, given as a discrete number and unit of measurement
☐	☒	A statement on whether measurements were taken from distinct samples or whether the same sample was measured repeatedly
☒	☐	The statistical test(s) used AND whether they are one- or two-sided<br>*Only common tests should be described solely by name; describe more complex techniques in the Methods section.*
☒	☐	A description of all covariates tested
☒	☐	A description of any assumptions or corrections, such as tests of normality and adjustment for multiple comparisons
☒	☐	A full description of the statistical parameters including central tendency (e.g. means) or other basic estimates (e.g. regression coefficient) AND variation (e.g. standard deviation) or associated estimates of uncertainty (e.g. confidence intervals)
☒	☐	For null hypothesis testing, the test statistic (e.g. *F*, *t*, *r*) with confidence intervals, effect sizes, degrees of freedom and *P* value noted<br>*Give P values as exact values whenever suitable.*
☒	☐	For Bayesian analysis, information on the choice of priors and Markov chain Monte Carlo settings
☒	☐	For hierarchical and complex designs, identification of the appropriate level for tests and full reporting of outcomes
☒	☐	Estimates of effect sizes (e.g. Cohen's *d*, Pearson's *r*), indicating how they were calculated

Our web collection on statistics for biologists contains articles on many of the points above.

Software and code

Policy information about availability of computer code

Data collection	Zen imaging software 2.6; Solution software (GC-MS QP2010 system)
Data analysis	Zen imaging software 2.6; Solution software (GC-MS QP2010 system); FastQC v0.11.9; cutadapt v3.4; BBMap v38.79; Bowtie2 v2.3.4.3; Langmead/Freebayes v1.0.0; bedtools v2.29.2; samtools/bcftools v1.9 using htslib 1.9; MapDamage; snpAD (v0.3.5d)

For manuscripts utilizing custom algorithms or software that are central to the research but not yet described in published literature, software must be made available to editors and reviewers. We strongly encourage code deposition in a community repository (e.g. GitHub). See the Nature Portfolio guidelines for submitting code & software for further information.

Data

Policy information about availability of data

All manuscripts must include a data availability statement. This statement should provide the following information, where applicable:

- Accession codes, unique identifiers, or web links for publicly available datasets
- A description of any restrictions on data availability
- For clinical datasets or third party data, please ensure that the statement adheres to our policy

Data relative to the present research have been provided in the text and in supplementary information.

Human research participants

Policy information about studies involving human research participants and Sex and Gender in Research.

Reporting on sex and gender	None
Population characteristics	None
Recruitment	None
Ethics oversight	None

Note that full information on the approval of the study protocol must also be provided in the manuscript.

Field-specific reporting

Please select the one below that is the best fit for your research. If you are not sure, read the appropriate sections before making your selection.

☐ Life sciences ☐ Behavioural & social sciences ☒ Ecological, evolutionary & environmental sciences

For a reference copy of the document with all sections, see nature.com/documents/nr-reporting-summary-flat.pdf

Ecological, evolutionary & environmental sciences study design

All studies must disclose on these points even when the disclosure is negative.

Study description	This work provides evidence about the role of edible plants for Italian early Neolithic individuals and relative cultural landscape.
Research sample	Dental calculi from human and animal specimens exhumed at Grotta Mora Cavorso (Lazio), one of the largest prehistoric burial deposits in central Italy, have returned an archaeobotanical record made up of several types of palaeoecological proxies. The organic fraction of this matrix was investigated by a multidisciplinary approach, whose novelty consisted in the application of next generation sequencing to ancient plant DNA fragments, specifically codifying for the maturase K barcode gene.
Sampling strategy	All samples available for the archeaological site were collected.
Data collection	Microscopic, chromatographic, and genetic analyses were carried out by the various authors of the work.
Timing and spatial scale	Samples were collected all together in October 2019.
Data exclusions	No data was excluded.
Reproducibility	Repetitions were successfull.
Randomization	No randomization occurred.
Blinding	All samples were tagged with numbers without specifying their origin.

Did the study involve field work? ☐ Yes ☒ No

Reporting for specific materials, systems and methods

We require information from authors about some types of materials, experimental systems and methods used in many studies. Here, indicate whether each material, system or method listed is relevant to your study. If you are not sure if a list item applies to your research, read the appropriate section before selecting a response.

Materials & experimental systems

n/a	Involved in the study
☒	☐ Antibodies
☒	☐ Eukaryotic cell lines
☐	☒ Palaeontology and archaeology
☒	☐ Animals and other organisms
☒	☐ Clinical data
☒	☐ Dual use research of concern

Methods

n/a	Involved in the study
☒	☐ ChIP-seq
☒	☐ Flow cytometry
☒	☐ MRI-based neuroimaging

Palaeontology and Archaeology

Specimen provenance	Specimens derived from the archeological site of Grotta Mora Cavorso (central Italy). The authorization to analyse these samples was provided by Prof. Mario Federico Rolfo of the Department of History, Culture and Society, University of Rome "Tor Vergata", Rome, Italy who is the responsible for them.
Specimen deposition	Department of History, Culture and Society, University of Rome "Tor Vergata", Rome, Italy
Dating methods	No new radiocarbon dating were produced. The calibration was obtained by OxCal (version 4.4.4), using the IntCal20 curve.

☒ Tick this box to confirm that the raw and calibrated dates are available in the paper or in Supplementary Information.

Ethics oversight	No ethical approval was required.

Note that full information on the approval of the study protocol must also be provided in the manuscript.
